# Counselling sessions increased duration of exclusive breastfeeding: a randomized clinical trial with adolescent mothers and grandmothers

**DOI:** 10.1186/1475-2891-13-73

**Published:** 2014-07-17

**Authors:** Luciana Dias de Oliveira, Elsa Regina Justo Giugliani, Lilian Córdova do Espírito Santo, Leandro Meirelles Nunes

**Affiliations:** 1 Department of Social Medicine, School of Medicine, Universidade Federal do Rio Grande do Sul (UFRGS), Ramiro Barcelos, 2400, bairro Santa Cecília, CEP 90035-003 Porto Alegre, RS, Brazil; 2 Centro de Estudos em Alimentação e Nutrição (CESAN), UFRGS, Ramiro Barcelos, 2400, bairro Santa Cecília, CEP 90035-003 Porto Alegre, RS, Brazil; 3 Post-Graduate Program in Children and Adolescent Health, UFRGS, Ramiro Barcelos, 2400, 2° andar, bairro Santa Cecília, CEP 90035-003 Porto Alegre, RS, Brazil; 4 Nursing School, UFRGS, Rua São Manoel, 963, bairro Rio Branco, CEP 90620-110 Porto Alegre, RS, Brazil; 5 Hospital de Clinicas de Porto Alegre, Ramiro Barcelos, 2350, bairro Santa Cecília, CEP 90035-903 Porto Alegre, RS, Brazil

**Keywords:** Clinical trial, Breastfeeding, Adolescent health, Family relations

## Abstract

**Background:**

Considering that adolescent mothers may be more vulnerable to discontinuing exclusive breastfeeding (EBF) before 6 months and that their mothers may exert a negative influence on this practice, this study was conducted with the objective of evaluating the efficacy of breastfeeding counselling for adolescent mothers and their mothers in increasing EBF duration.

**Methods:**

A clinical trial was performed in 323 adolescent mothers with newborns and their mothers randomized in four groups: (1) not living with mother, without intervention; (2) not living with mother, with intervention; (3) living with mother, without intervention, (4) living with mother, with intervention. The intervention consisted of five counselling sessions directed to mother and grandmother, in the maternity hospital and on follow-up. Information about feeding practices during the newborn’s first six months of life was collected monthly by telephone. Intervention’s efficacy was measured through Cox regression and comparison of exclusive breastfeeding medians and survival curves for the different groups.

**Results:**

The intervention increased the duration of EBF by67 days for the group which included grandmothers (HR = 0.64; CI 95% = 0.46-0.90) and 46 days for the group which did not include grandmothers (HR = 0.52; CI 95% = 0.36-0.76).

**Conclusions:**

Counselling sessions in the first four months of children’s lives proved to be effective in increasing EBF duration among adolescent mothers.

**Trial registration:**

ClinicalTrials.gov NCT00910377.

## Background

The contribution of exclusive breastfeeding (EBF) to the decrease in infant morbidity and mortality has already been well documented. EBF in the first 6 months of life, as recommended by the World Health Organization (WHO) [1], protects against gastrointestinal infections [2-4], respiratory infections [5,6], allergies [7], and non-communicable chronic diseases [8]. Despite these benefits, EBF rates are still very low [8]. In Brazil, although the rates have increased in the last decades, the prevalence of EBF in babies under 6 months of age is 41%, with a median duration of 54 days [9].

Studies in different countries have identified various factors associated with the early abandonment of EBF [10,11] among young mothers [12-17]. In Brazil, it was found that adolescent mothers had a 1.5 times greater risk of abandoning EBF before their babies were 6 months of age when compared with adult women [17]. Low socioeconomic status [18-22], difficulties with breastfeeding like pain [23], sore nipple and mastitis [18,19], the presence of a partner [19] and negative familial influence [18,19,22,24] are all factors associated with the early abandonment of EBF in young mothers.

Among the people who exert influence over the adolescent mother is her mother (the maternal grandmother of the child); she frequently and actively participates in the decisions related to the feeding of her grandchild. Her experience and personal opinion about breastfeeding may facilitate or hamper this practice [25]. In Brazil, there are at least 3 studies indicating that the duration of EBF may be less due to a grandmother’s influence, especially from the maternal grandmother [26-28].

The finding that adolescent mothers are more vulnerable to discontinuing EBF before 6 months and that their mothers may exert a negative influence on EBF reinforces the importance of strategies directed at this segment of the population to promote, protect and support EBF.

A systematic review that included 52 articles from 21 countries concluded that all forms of extra support for breastfeeding mothers by both lay and professionals had a positive impact on breastfeeding outcomes, especially strategies that rely mainly on face-to-face support [29]. The few studies about the influence of support to adolescent mothers show some positive effect too [30,31]. To our knowledge, no studies have evaluated breastfeeding support directed to boths teenage mothers and grandmothers.

Thus, this study was conducted with the objective of evaluating the efficacy of multiple breastfeeding counselling sessions with adolescent mothers and their own mothers in preventing the early abandonment of EBF.

## Methods

A randomized clinical trial involving adolescent mothers, their babies and their mothers (maternal grandmothers) who lived together in the same house and were attended at the maternity clinic of the Hospital de Clínicas de Porto Alegre was conducted. This general university hospital is accredited as a “Baby-Friendly Hospital” and performs between 3,000 and 4,000 births a year. The majority of the population assisted at this hospital is patients of low socioeconomic status who use the public health care system.

To calculate the sample size, the following parameters were adopted: α = 5%; β = 20%; ratio exposed/not exposed = 1:1; prevalence of EBF in the first month in the group not exposed to the intervention = 56% [32], and difference in the prevalence of EBF in the first month between the exposed group and group not exposed to the intervention = 25 percentage points. Thus, a minimum number of 48 subjects in each group were evaluated, to which 50% more subjects were added to compensate for possible losses and to facilitate the performance of multivariate analyses, totalling approximately 72 participants in each group.

Initially, a pilot study with 20 mothers was conducted to test the data collection instruments. To ensure that the intervention attended to the interests of the mothers and grandmothers, meetings with the adolescent mothers and the maternal grandmothers were conducted before beginning the study; these meetings helped to define the content and the intervention approach.

The adolescent mothers were recruited in the period from May 2006 to January 2008 and the follow-up occurred from June 2006 to February 2009. Daily, including on the weekends, adolescent mothers (younger than 20 years of age) who did and did not live with their own mothers were identified in the obstetric inpatient unit and screened for the fulfilment of the following inclusion criteria: living in the city of Porto Alegre, with healthy non-twin newborn infants, in the rooming-in ward, having started breastfeeding, with infant birth weight greater than or equal to 2,500 g. Pairs who had to be separated due to problems related to the mother or the baby were excluded from the study; additionally, adolescents who lived with their newborns’ paternal grandmother were excluded.

Once identified, the mothers were allocated into two groups: those who lived with their own mothers (the babies’ maternal grandmothers) and those who did not. These two groups were then randomly subdivided into blocks of two subjects for control or intervention groups; that is, if a mother was selected to the control group, the next eligible mother would necessarily be included in the intervention group. At the end, there were a total of four study groups, namely: (1) adolescent mothers not living with their own mothers and not exposed to the study intervention; (2) adolescent mothers not living with their own mothers but exposed to the study intervention; (3) adolescent mothers still living with their own mothers and not exposed to the study intervention; and (4) adolescent mothers still living with their own mothers and exposed to an intervention program directed at both.

Two researchers were specially hired to perform the selection of the mothers and administer the intervention. In the intervention groups, when the mothers and grandmothers lived together, both participated in the intervention. When they did not live together, only the mother received the intervention. The inclusion of this last group sought to evaluate to what extent the impact of intervention could be attributed solely to the adolescent.

Data collection was conducted at distinct periods. In the maternity hospital, after agreeing to participate in the study and signing an informed consent form, the adolescent mothers and the maternal grandmothers responded, through an interview, a structured questionnaire to obtain sociodeomographic data and information related to pre-natal care, the birth, and their previous experience with breastfeeding. Information about how the babies were fed in their first 6 months of life was obtained monthly, in a telephone interview with the mother, using a structured questionnaire containing questions about the child’s feeding. If telephone contact could not be made, in-house visits performed by research assistants who were blinded to the intervention. To verify the quality of the information, 5% of the mothers were randomly selected each month to undergo a second interview with the principal field investigator; this interview focused on some key questions from the follow-up survey.

The intervention sessions occurred at the maternity hospital and at the mothers’ homes. In the hospital, right before the time of discharge, breastfeeding counselling session were given in accordance with WHO guiding principles [33], that is, mother and professional dialoguing about many aspects of breastfeeding, such as breastfeeding importance and duration, factors that interfere with the production of milk, the technique of breastfeeding, the consequences of using a pacifier and the baby’s crying and communication. Doubts specific to each mother and grandmother were addressed and clarified as well. The mothers were encouraged to breastfeed during the intervention, whenever possible, to take advantage of the opportunity to observe the breastfeeding technique and to instruct as to the positioning of mother/baby and latch on.

The sessions were conducted by one member of the team formed by 2 nurses, a nutritionist and a paediatrician. All members had ample experience with breastfeeding; 3 were International Board Certified Lactation Consultant (IBCLC). These interventions were conducted individually and separately with each mother and grandmother. At home, the counselling sessions were conducted simultaneously.

For support material, flipcharts specially created for the study were used; one flipchart was prepared for the adolescent mother and another for the grandmother. These tools contained information about breastfeeding and about the prevention and management of the most common problems. One booklet with content similar to what was in the flipcharts was distributed at the end of the counselling session in the maternity hospital. In the booklet, there was space to add photos of the baby, which were obtained by the professional during the home visits.

In the home, when the mothers and grandmothers lived together, they received joint counselling sessions when the infants were 7, 15, 30, 60 and 120 days of age. In these sessions, the team members discussed difficulties confronted by the mothers and how they managed them and reinforced the messages given at the counselling sessions in the maternity hospital. Since many mothers in Brazil introduce complementary feeding when the children turn 4 months, this issue was discussed in the visit of the fourth month, emphasizing the importance of EBF in the first six months of life.

The expected outcome was the likelihood of children being exclusively breastfed at 6 months of life. EBF was considered to occur when the infants received breast milk as their only source of nutrition and hydration, without any solid or liquid supplement, including water and tea [1].

The database for the study was assembled using Microsoft Excel, with double entry of data. Statistical analysis was performed with SPSS 16.0.

The analyses were based upon the “intention to treat” principle. Initially, the characteristics of the control and intervention group participants were compared using the chi-squared test with Yates’ correction. The EBF median duration was calculated, along with their respective confidence intervals of 95%, in days; the Kaplan-Meier survival curves were constructed for EBF in the first 6 months for the 4 groups. To test the difference between the curves, the log-rank test was used. To quantify the impact of the intervention in the abandonment of EBF in the first 6 months, Cox’s regression was used via the hazard-ratio and its respective confidence interval of 95%.

The study was approved by the Ethics and Research Committee of the Hospital de Clínicas de Porto Alegre. The study was registered at ClinicalTrials.gov under the number NCT00910377.

## Results

**Figure 1:**
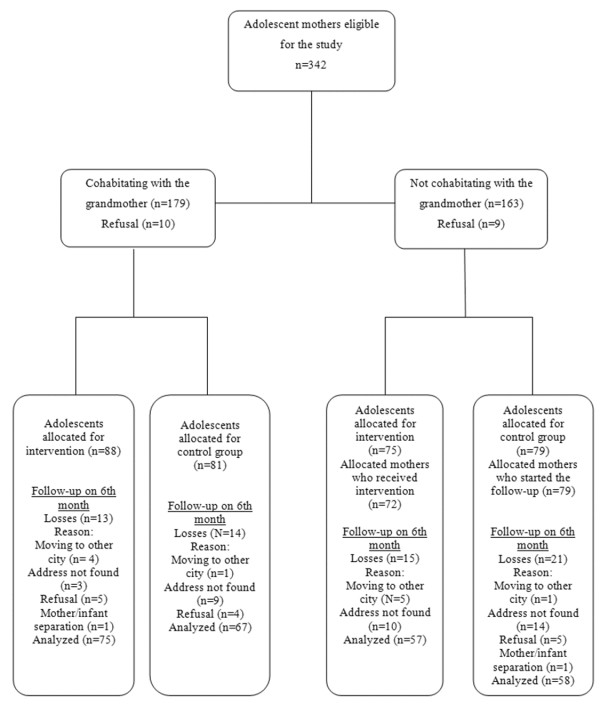
Flowchart of randomization of the study since the beginning of the collection until the sixth month of follow-up.

Figure 1 presents the clinical trial profile from recruitment of the individuals until the last outcome assessment, when the infants had completed 6 months of life. Of the 342 adolescent mothers eligible for the study, 19 (5.5%) were not included due to their refusal to participate. After randomization, there were 66 losses during the study (intervention: n = 31/163 – 19.0%; controls: 35/160 – 21.9%).

Table 1 shows that there was a balance in the distribution of the basic characteristics between the components of the control and intervention groups.

**Table 1:** Characteristics of adolescent mothers, their infants and maternal grandmother, by the intervention exposure*

**Infant and mother characteristics**	**Intervention (n = 163)**	**Control (n = 160)**
Infant gender, Masculine: n (%)	76 (46.6)	88 (55.0)
Birth weight: median ± 2 SD (g)	3,280.50 (406.9)	3,205.93 (398.0)
Breastfeeding duration ≥6 months of previous children**: n (%)	13/22 (59.0)	11/25 (44.0)
Maternal schooling level > 8 years: n (%)	44 (27.0)	37 (23.1)
Mothers living with a partner: n (%)	102 (62.6)	100 (62.5)
≥7 prenatal care visits: n (%)	106 (65.0)	97 (60.6)
Primiparity: n (%)	141 (86.5)	135 (84.4)
Vaginal delivery: n (%)	120 (73.6)	121 (75.6)
**Grandmothers characteristics**	**Intervention(n = 88)**	**Control(n = 81)**
Grandmother age: mean ± 2 SD (years)	43.9 (7.4)	45.1 (8.4)
Grandmother working outside of the home: n (%)	51 (63.7)	56 (63.6)
Grandmother schooling level > 8 years: n (%)	21 (24.1)	13 (16.5)
Breastfeeding duration for the adolescent mothers: mean ± 2 SD (months)	17.7 (18.7)	12.9 (16.5)

*p > 0.05 for all variants; **primiparous excluded (n intervention =22; n control = 25).

**Figure 2:**
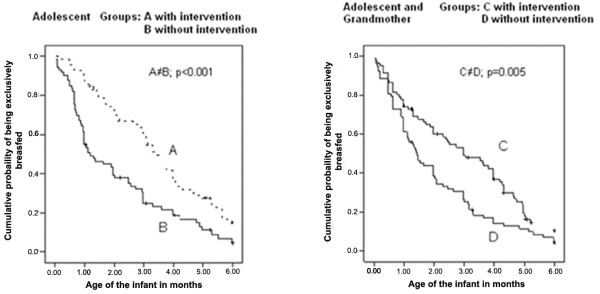
Kaplan-Meier curve of exclusive breastfeeding in the first 6 months of living in groups of adolescent mothers without the presence of maternal grandmother, intervention (A) and control (B) and in groups with the presence of the maternal grandmother, intervention (C) and control (D).

**Figure 3:**
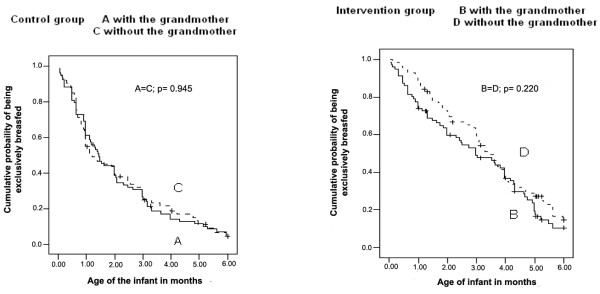
Kaplan-Meier curve of EB in the first 6 months of life in control group (Aand C) and intervention group (B and D), according to cohabitation with the maternalgrandmother.

The calculation of the medians indicated an increase in the duration of EBF in the 2 intervention groups in relation to the groups without intervention. The intervention increased by 67daysthe duration of EBFin the group of adolescents not living with their mothers (103 days; CI 95% = 82.4-123.5 with intervention versus 36 days; CI 95% = 21.5 – 50.5 without intervention) and by 46 days in the group of mothers living with the grandmothers (89 days; CI 95% = 56.8-121.2 with intervention versus 43 days; CI 95% = 29.5-56.1 without intervention). Considering only the control and intervention groups, without taking into account the mother’s cohabitation with the maternal grandmother, the median duration of EBF were, respectively, 41 days (CI 95% = 30.5-51.5) and 99 days (CI 95% = 83.4-114.6).Figure 2 presents the survival curves for EBF in the first 6 months of the infants’ lives for the control and interventions groups, with and without inclusion of the maternal grandmother in the intervention. In both situations, the prevalence of EBF were significantly greater in the groups that received the intervention. Cox’s regression estimated that the intervention, when applied only to the adolescent mothers, reduced by 48% the abandonment of EBF in the first 6 months (HR [hazard ratio] = 0.52; CI 95% = 0.36-0.76), and when the maternal grandmothers lived with the adolescent, the intervention reduced abandonment of EBF by 36% (HR = 0.64; CI 95% = 0.46-0.90).The influence of the grandmothers’ presence on the prevalence of EBF in the first 6 months can be seen in the survival curves presented in Figure 3. Although the EBF frequencies at various points on the curve were greater in the group that received the intervention when the grandmother did not live with the mother, there was no significant statistical difference in these frequencies, independent of whether or not the grandmother resided in the same home.

## Discussion

The increasing EBF rates trends in Brazil is undeniable. In 1986, only 3.6% of infants between 0-4 months were exclusively breastfeed [32]; in 2008, this prevalence rose to 51.2% [9]. However, the median duration of this practice in Brazil is still very low, not yet reaching 2 months. The last national survey (2008) clearly showed that in the places where the prevalence of EBF were higher 10 years ago, the advances were more discreet and there were even setbacks [9]. This scenario indicates that, in order to reach better EBF rates, it is necessary to invest in new strategies promoting breastfeeding, especially in the groups most likely to abandon this practice. The last national survey confirmed that adolescent mothers constitute an at-risk group for the early abandonment of EBF. The prevalence of EBF in infants under 6 months of age was 44.4% for those whose mothers were between 20 and 35 years, and it was35.8% for infants of adolescent mothers [9].

This randomized clinical trial showed that it is possible, with systematic counselling directed at adolescent mothers initiated at the maternity hospital and maintained in the first 4 months, to increase more than 2 months the duration of EBF and to reduce by 48% the abandonment of this practice before 6 months among adolescent mothers. This finding is significant because, with the increase in the duration of EBF from 36 days to 103 days, the protection of children against diseases and death increases substantially, as previous studies have demonstrated. One of these studies, conducted in the United Kingdom, estimated that each month of EBF may prevent an additional 53% of the hospitalizations due to diarrhoea and 27% of the hospitalizations due to respiratory tract diseases [34]. In Brazil, it was demonstrated that the chance of hospitalization due to pneumonia in the first 3 months was 61 times greater in children not breastfed in comparison to those who were exclusively breastfed [5]. Also, it is worthy to mention that the increase in the duration of EBF due to the intervention was 2 times bigger than the increase experienced in Brazil in the last decade (67 days *versus* 30 days) [9].

There are few studies that have tested strategies of EBF promotion directed at adolescent mothers. In Canada, phone calls encouraging breastfeeding by adolescent mothers who breastfed demonstrated efficacy in increasing the duration of EBF in this population [35], which strengthened the conclusion that adolescent mothers were susceptible to changes related to healthier feeding practices for their babies.

The decision to include maternal grandmothers who live with the adolescent mothers in the intervention arose due to findings from previous studies by the authors of this work [27,28]. These findings indicated that grandmothers, especially the maternal ones, may negatively influence the duration of EBF. The results of this study, however, did not confirm this suspicion, as there was no difference in the prevalence of EBF among mothers who lived or did not live with their mothers in both the control and intervention groups. Methodological differences may explain this apparent disagreement as the previous studies were restricted in their evaluation of the abandonment of EBF in the first month of life, and the samples were made up of women of all ages. In the present study, we evaluated EBF during the first six months of child’s life and only among adolescent mother. However, it is important to mention that despite the absence of a significant statistical difference, the impact of intervention when the mother lives with the grandmother was lower than that shown in the group in which the mother did not live with the grandmother (HR 0.52 versus 0.76). Curiously, in the group that underwent intervention, the prevalence of EBF at various curve points were higher when the grandmother was not present, suggesting a modest negative influence of the grandmothers on the rates of EBF among adolescent mothers. It is possible that an adolescent mother who does not live with her mother has more autonomy and follows the instructions of the health professional more easily with regard to feeding her baby. Also, it is possible that adolescent mothers who live with their mothers share the responsibility to care for the children, giving young mothers the opportunity to go out to study, work or socialize with friends or boyfriends, possibly to the detriment of EBF duration.

This study has the distinction of being, as far as we know, the first to evaluate the impact of educational actions in the duration of EBF using a randomized clinical trial in a population considered to be at risk for the early abandonment of this practice: adolescent mothers living with the maternal grandmothers of their children. However, caution is necessary in generalizing these results because it is an efficacy study that has been conducted in only one setting. It is prudent to confirm these findings in different populations and in conditions mimicking the reality of each locale (effectiveness studies). In Brazil, for example, this strategy can be tested with the Family Health Strategy teams. In this strategy, which covers 51% of the Brazilian population, teams of various professionals provide care for families of a determined geographical area, performing actions to promote and maintain the health in the community, including in the home.

## Conclusions

Multiple breastfeeding counselling sessions with adolescent mothers and their own mothers, when they lived together, proved to be effective in increasing EBF duration. It postponed the abandonment of EBF to beyond the third month of life. Therefore, this strategy has the potential to save many lives, avoid hospitalizations and give better quality of life and health of children from adolescent mothers.

## Abbreviations

EBF: Exclusive breastfeeding; IBCLC: International Board Certified Lactation Consultant; WHO: World Health Organization.

## Competing interests

The authors declare that they have no competing interests.

## Authors’ contributions

LDO and ERJG participated in the study concept and design, analysis and interpretation of data, drafting of the manuscript and critical revision for important intellectual content. All authors contributed to the final version of the manuscript and participated in the acquisition of data and in administrative, technical, and material support. All authors read and approved the final manuscript.

## Authors’ information

Luciana Dias de Oliveira – PhD, Professor at the School of Nutrition of Universidade Federal do Rio Grande do Sul, Department of Social Medicine.

Elsa Regina Justo Giugliani –MD, PhD, Full Professor at the Department of Pediatrics of the School of Medicine at Universidade Federal do Rio Grande do Sul.

Lilian Cordova do Espírito Santo – RN, PhD, Professor at the School of Nurse of Universidade Federal do Rio Grande do Sul.

Leandro Meirelles Nunes – MD, MsC, PhD student of the postgraduate course in Child Helth of the School of Medicine of Universidade Federal do Rio Grande do Sul.

## References

[B1] Organization WH Global strategy for infant and young child feeding. the optimal duration of exclusive breastfeeding http://apps.who.int/gb/archive/pdf_files/WHA54/ea54id4.pdf

[B2] Duijts L Ramadhani MK Moll HA Breastfeeding protects against infectious diseases during infancy in industrialized countries. a systematic review Matern Child Nutr 2009 5 199 210 19531047 10.1111/j.1740-8709.2008.00176.x PMC6860885

[B3] Bhandari N Bahl R Mazumdar S Martines J Black RE Bhan MK Infant Feeding Study G Effect of community-based promotion of exclusive breastfeeding on diarrhoeal illness and growth: a cluster randomised controlled trial Lancet 2003 361 1418 1423 12727395 10.1016/S0140-6736(03)13134-0

[B4] Kramer MS Kakuma R The optimal duration of exclusive breastfeeding: a systematic review Adv Exp Med Biol 2004 554 63 77 15384567 10.1007/978-1-4757-4242-8_7

[B5] César JA Victora CG Barros FC Santos IS Flores JA Impact of breast feeding on admission for pneumonia during postneonatal period in Brazil: nested case-control study BMJ 1999 318 1316 1320 10323815 10.1136/bmj.318.7194.1316 PMC27869

[B6] Chantry CJ Howard CR Auinger P Full breastfeeding duration and associated decrease in respiratory tract infection in US children Pediatrics 2006 117 425 432 16452362 10.1542/peds.2004-2283

[B7] van Odijk J Kull I Borres MP Brandtzaeg P Edberg U Hanson LA Host A Kuitunen M Olsen SF Skerfving S Sundell J Wille S Breastfeeding and allergic disease: a multidisciplinary review of the literature (1966-2001) on the mode of early feeding in infancy and its impact on later atopic manifestations Allergy 2003 58 833 843 12911410 10.1034/j.1398-9995.2003.00264.x

[B8] Horta BL Bahl R Martines J Victora CG Evidence on the long term effects of breastfeeding. Systematic reviews and meta-analyses 2007 Geneva, Switzerland: World Health Organization

[B9] Venancio SI Escuder MM Saldiva SR Giugliani ER Breastfeeding practice in the Brazilian capital cities and the Federal District: current status and advances J Pediatr (Rio J) 2010 86 317 324 20711546 10.2223/JPED.2016

[B10] Duong DV Lee AH Binns CW Determinants of breast-feeding within the first 6 months post-partum in rural Vietnam J Paediatr Child Health 2005 41 338 343 16014137 10.1111/j.1440-1754.2005.00627.x

[B11] Parizoto GM Parada CMGL Venâncio SI Carvalhaes MABL Tendência e determinantes do aleitamento materno exclusivo em crianças menores de 6 meses J Pediatr (Rio J) 2009 85 201 208 19492168 10.2223/JPED.1888

[B12] Dubois L Girard M Social determinants of initiation, duration and exclusivity of breastfeeding at the population level: the results of the Longitudinal Study of Child Development in Quebec (ELDEQ 1998–2002 Can J Public Health 2003 94 300 305 12873091 10.1007/BF03403610 PMC6979909

[B13] Bueno MB de Souza JM de Souza SB da Paz SM Gimeno SG de Siqueira AA [Risks associated with the weaning process in children born in a university hospital: a prospective cohort in the first year of life, Sao Paulo, 1998-1999 Cad Saude Publica 2003 19 1453 1460 14666227 10.1590/s0102-311x2003000500024

[B14] Venancio SI Monteiro CA Individual and contextual determinants of exclusive breast-feeding in Sao Paulo, Brazil: a multilevel analysis Public Health Nutr 2006 9 40 46 16480532 10.1079/phn2005760

[B15] Lande B Andersen LF Baerug A Trygg KU Lund-Larsen K Veierod MB Bjorneboe GE Infant feeding practices and associated factors in the first six months of life: the Norwegian infant nutrition survey Acta Paediatr 2003 92 152 161 12710639 10.1111/j.1651-2227.2003.tb00519.x

[B16] Venancio SI Escuder MML Kitoko P Rea MF Monteiro CA Freqüência e determinantes do aleitamento materno em municípios do Estado de São Paulo Rev Saude Publica 2002 36 313 318 12131970 10.1590/s0034-89102002000300009

[B17] Santo LC de Oliveira LD Giugliani ER Factors associated with low incidence of exclusive breastfeeding for the first 6 months Birth 2007 34 212 219 17718871 10.1111/j.1523-536X.2007.00173.x

[B18] Sepka GC Gasparelo L Silva ABF Mascarenhas TT Promoção do aleitamento materno com mães adolescentes: acompanhando e avaliando essa prática Cogitare Enferm 2007 12 313 22

[B19] Frota DA Marcopito LF Breastfeeding among teenage and adult mothers in Brazil Rev Saude Publica 2004 38 85 92 14963546 10.1590/s0034-89102004000100012

[B20] Park YK Meier ER Song WO Characteristics of teenage mothers and predictors of breastfeeding initiation in the Michigan WIC Program in 1995. Women, Infants, and Children J Hum Lact 2003 19 50 56 12587645 10.1177/0890334402239734

[B21] Misra R James DC Breast-feeding practices among adolescent and adult mothers in the Missouri WIC population J Am Diet Assoc 2000 100 1071 1073 11019358 10.1016/S0002-8223(00)00312-6

[B22] Wiemann CM DuBois JC Berenson AB Racial/ethnic differences in the decision to breastfeed among adolescent mothers Pediatrics 1998 101 E11 9606253 10.1542/peds.101.6.e11

[B23] Dykes F Moran VH Burt S Edwards J Adolescent mothers and breastfeeding: experiences and support needs–an exploratory study J Hum Lact 2003 19 391 401 14620453 10.1177/0890334403257562

[B24] Ineichen B Pierce M Lawrenson R Teenage mothers as breastfeeders: attitudes and behaviour J Adolesc 1997 20 505 509 9368128 10.1006/jado.1997.0105

[B25] Grossman LK Harter C Sachs L Kay A THe effect of postpartum lactation counseling on the duration of breast-feeding in low-income women Am J Dis Child 1990 144 471 474 2321612 10.1001/archpedi.1990.02150280093019

[B26] Andrade IG Taddei JA Social-economical, cultural and family determinants in the city of Natal, Brazil Rev Paul Pediatr 2002 20 8 16

[B27] Susin LR Giugliani ER Kummer SC Influence of grandmothers on breastfeeding practices Rev Saude Publica 2005 39 141 147 15895130 10.1590/s0034-89102005000200001

[B28] Giugliani ER do Espirito Santo LC de Oliveira LD Aerts D Intake of water, herbal teas and non-breast milks during the first month of life: associated factors and impact on breastfeeding duration Early Hum Dev 2008 84 305 310 17888592 10.1016/j.earlhumdev.2007.08.001

[B29] Renfrew MJ McCormick FM Wade A Quinn B Dowswell T Support for healthy breastfeeding mothers with healthy term babies Cochrane Database Syst Rev 2012 16 5 CD001141. doi:10.1002/14651858 10.1002/14651858.CD001141.pub4 PMC3966266 22592675

[B30] Wambach CA Aaronson L Breedlove G Domian EW Rojjanasrirat W Yeh HW A randomized controlled trial of breastfeeding support and education for adolescent mothers West J Nurs Res 2011 33 486 505 doi:10.1177/0193945910380408 20876551 10.1177/0193945910380408

[B31] Meglio GD McDermott MP Klein JD A randomized controlled trial of telephone peer support’s influence on breastfeeding duration in adolescent mothers Breastfeed Med 2010 5 41 47 20043705 10.1089/bfm.2009.0016

[B32] Braun ML Giugliani ER Soares ME Giugliani C de Oliveira AP Danelon CM Evaluation of the impact of the baby-friendly hospital initiative on rates of breastfeeding Am J Public Health 2003 93 1277 1279 12893612 10.2105/ajph.93.8.1277 PMC1447954

[B33] Organization/UNICEF WH Breastfeeding counselling: a training course 1993 Geneva, Switzerland: World Health Organization/UNICEF

[B34] Quigley MA Kelly YJ Sacker A Breastfeeding and hospitalization for diarrheal and respiratory infection in the United Kingdom Millennium Cohort Study Pediatrics 2007 119 e837 e842 17403827 10.1542/peds.2006-2256

[B35] DATASUS BMdS Estatísticas vitais http://www2.datasus.gov.br/DATASUS/index.php?area=0205

